# The role of chess in the development of children-parents’ perspectives

**DOI:** 10.3389/fpsyg.2023.1210917

**Published:** 2023-06-26

**Authors:** Costica Ciprian Nanu, Claudiu Coman, Maria Cristina Bularca, Luiza Mesesan-Schmitz, Mihaela Gotea, Ioana Atudorei, Ioan Turcu, Ion Negrila

**Affiliations:** ^1^Doctoral School of Social and Humanities Sciences, University of Craiova, Craiova, Romania; ^2^Department of Social Sciences and Communication, Transilvania University of Brasov, Brasov, Romania; ^3^Department of Motor Performance, Transilvania University of Brasov, Brasov, Romania

**Keywords:** chess, parents, children, benefits, development

## Abstract

**Introduction:**

The study examines the role of chess in the development of children from the perspectives of parents. The research focused on analyzing the parents’ perceptions about chess’s role in their children’s development, on finding out how the perception of parents differs depending on whether they know how to play chess or not, and on outlining the profile of the parents whose children play chess.The study was conducted in Romania.

**Methods:**

In order to conduct the study, a quantitative research method was used, while having as a research instrument a non-standardized questionnaire. The questionnaire was applied to parents of chess-playing children who are members of chess clubs from Romania. The sample of the study comprises 774 respondents.

**Results:**

The results of our research showed that parents are of the opinion that chess helps children develop their cognitive abilities, their character and their competitive spirit. Most of the parents focused on highlighting the positive effects of chess on the development of their children. Parents also considered that chess helped their children develop positive emotions and helped them overcome negative emotions. The results revealed differences between the opinions of parents depending on whether they know how to play chess or not. Thus, parents who do know how to play chess were more likely to focus on the positive effects of the game on the development of their children, and those who know how to play chess were also more satisfied with their children’s accumulated knowledge following chess lessons.

**Discussion:**

Findings extend our understanding of how parents perceive the way chess influences the development of their children, it offered us a perspective on the perceived benefits of chess, benefits which should be further analyzed in order to identify under what circumstances chess could be introduced in the school curriculum.

## 1. Introduction

In post-modern societies, concerns for the quality of offspring are widespread among parents compared to other macrosocial contexts. Nowadays, research related to optimal child development is a traditional and yet topical, interdisciplinary field, concerned with the internal and external factors that help children develop and improve their ability to become successful youth and adults, to conduct to a successful life.

Regarding the external factors, we can underline that social environment comprises important aspects that support the understanding of child development process. Theories of human development that take into account the social-cultural influences on child development process are connected with authors like Vygotsky (1978) and Bronfenbrenner (1979). We consider that these theories are adequate to analyze our topic, because parents shape the child’s first developmental environment, characterized by significant interactions and influences, over a long period of time.

According to sociocultural theory of child development of Vygotsky (1978), human learning is primarily a social process and the initiation of human intelligence takes place in society or culture. Vygotsky (1978) considers that cultural development (as opposed to natural development) is associated with language and reasoning capability. This theory focus on the interaction between individuals and the culture in their environment, interaction which exerts an important role in children’s cognitive development. In perspective of Vygotsky (1978), the learning process can be synthetized as follow: in the first phase children interact with others, then they integrate the obtained data from the social interaction into their mental structure.

Chess, beyond the rules that it implies and the game strategies used, is a continuous interaction between the two players, as well as with the immediate environment in which the competition is taking place.

The ecological systems theory, developed by Bronfenbrenner (1979), focuses and underlines the importance of the environment in children’s lives and development, stressing person-context interrelatedness. Child development and parent–child interactions (microsystem) are embedded in a mesosystem of a broader contexts (nuclear family, school, peers, neighborhood, and so on). Family life shapes and is shaped by the exosystem influences (extended family, workplace, mass media, health services, and neighbors). The macrosystem of culture, laws, values, and social classes shape and determine the immediate context of a child, the goals that parent have for their children, and the practices which they choose to achieve these goals.

During the process of child development, an important role is played by the parents, as agents of primary socialization. Parenting is a multidimensional concept that refers to how individuals raise their children, which includes attitudes (cognitions, feelings, and behavioral intentions) and practices regarding child-rearing. Parenting is a source of ambivalent feelings and experiences. Sometimes it is a source of deep satisfaction for both parent and child, and other times the quotidian responsibilities of being a parent generates inter-roles conflicts, stress, frustrations, and negative overwhelming emotions. But the crucial importance of parenting is widespread, parenting decisions and practices influence to a great extent the child’s outcomes.

Parents’ beliefs in general and their knowledge about child development in particular could guide their interactions with children and for their children. And they might be more likely to create an environment that is appropriate to develop their children’s emerging abilities. The analysis of parents’ perceptions about adequate activities for child development, such as chess, offers the opportunity to contour parents’ thoughts about desirable outcomes for children, as well as their role as parents. Parents who consider a particular activity to be important for child development may be more likely to seek out information about that area, make decisions, and act accordingly. Furthermore, the motivation of conducting such a study also comes from the lack of research conducted in Romania regarding the effect of chess at the level of children’ development. Thus, the authors believe that chess is a sport, because it manages to attract every shred of individual capacity and power, both physical and psychological, from and individual and ultimately involves competition. Moreover, chess is based on long-term research and analysis of principles that govern the process of finding deep moves that often hide complex reasoning, and chess could also be seen as an art, because it presents the intuition that it is a decision-making factor, this factor intervening when two moves have similar and sometimes contradictory assessments, being necessary for decision-making.

Parental ethnotheories are shared beliefs about the goals of child development and the socialization practices that will achieve these goals, according to Greenfield and Keller (2004). They generate specific parenting behaviors that, in turn, contribute to create the cultural adult. These ideas about childcare practices, as defined by a specific cultural environment, are related to specific developmental goals: for example, there are ethnotheories about sibling relationships (Kapısız and Sieben, 2022), about healthy eating (Srivastava et al., 2018), or about play and learning (Avornyo and Baker, 2022). Parental ethnotheories link socialization practices to cultural values and they transmit the socialization agendas of one generation to the next.

Parental ethnotheories constitute the third component of the child’s developmental niche (Super and Harkness, 1986). The other dimensions are: physical and social settings, and also childcare customs and practices.

Harkness and Super (1996) consider that these culturally constructed ideas are influenced by the larger environments in which families live, and they are important in parents’ decision making process regarding children’s settings of daily life as well as customs of care.

As part of a system of ideas and practices that reflect wider cultural models, parents’ cultural beliefs about a particular activity—such as playing chess—serve as guide to parenting practices, where playing chess messages are conveyed and experienced and, ultimately, generate children’s developmental outcomes. In other words, encouraging and supporting children to learn chess is a practice that can be shaped by parental ethnotheories, with the aim of developing some specific traits especially in cognitive, socio-emotional, and moral areas.

In the scientific literature, the list of domains of child development can contain several components. For example, in the contents of the Cambridge Encyclopedia of Child Development, published by Hopkins et al. (2017), there are the following domains: physical, perceptual and cognitive, language and communication, and social, emotional, and moral development. The same areas of child development are reflected in the chapters of The Sage Handbook of Developmental Psychology and Early Childhood Education written by Whitebread et al. (2019) and in the book written by Keenan et al. (2016). In all child development domains, parents play an important role, as they transmit specific cultural beliefs, preferences, behavioral patterns, make decisions, and offer social support for different activities.

Playing games with rules begins in late preschool and develops through school, according to Vygotsky (1978), and every game with rules constructs an imaginary situation. The game of chess, in particular, creates imaginary contexts because the game is governed by certain rules (for example, each piece can only move in a specific way) and a large number of possibilities for action are excluded. Imaginative play generates learning opportunities that could contribute to the child’s further development.

Research studies on chess have considered different categories of participants in collecting data. Some researchers have aimed to study specific age groups such as: preschool children (6 year old children—Gunes and Tugrul, 2017) primary school children (Bilalić et al., 2007b), adolescents and high school players (Melamed and Berman, 1981; Atashafrouz, 2019; Afshari et al., 2022), college players (Subia et al., 2019), adults (Blanch, 2018), and elderly people (Vale et al., 2018).

Others have done research on chess players with different performance levels, with focus on amateur/novice (Blanch and Llaveria, 2021), on professional players/experts (adolescent elite chess players—De Bruin et al., 2007; adult international chess grandmaster—Fuentes-García et al., 2022) or comparative studies on these categories, that provide more convincing evidences that chess master players are different from novice players in brain functional organization of local connections and global topologies (Song et al., 2022). This type of research focuses on the analysis of the conditions that lead to chess performance, including certain psycho-social and physical characteristics of the player, which we will detail later.

The game of chess was also described in several studies as a tool used in intervention programs for improving the outcomes and the quality of life for specific categories of people, especially vulnerable ones. Some studies report the results from chess training programs implemented in educational settings (Jerrim et al., 2016; Jankovic and Novak, 2019), or the effectiveness of playing chess as a treatment option for children with ADHD (ElDaou and El-Shamieh, 2015; Blasco-Fontecilla et al., 2016). This type of intervention programs was also used with the aim of improving general cognitive status, attention, processing speed, and executive functions in a sample of semi-institutionalized and institutionalized senior people (Cibeira et al., 2021), for the elderly with dementia (Wahyu Laksono et al., 2019), or to evaluate the benefits of chess in mathematics lessons for children with learning disabilities (Scholz et al., 2008).

Some researchers have examined the subjective perspective toward the benefits of chess, such as teachers’ perceptions; for example, Chitiyo et al. (2021) investigated the experiences and perceptions of teachers who used chess during the instruction process, and they found out that their participants have largely positive perception regarding chess and they perceived consistent benefits of chess among their students. Another type of perception consists in students’ perceived benefits of chess, explored by Chitiyo et al. (2023), who also examined how these benefits differ by gender and grade level.

As far as it is known, no previous study has analyzed the parents’ perceptions, beliefs, or ethnotheories about the role of playing chess in child development and our study aims to accomplish that.

At the level of common sense knowledge, there is a number of assumptions related to the benefits of chess on child development, such as: “Chess increases mathematical abilities”; “Chess improves academic performance”; “Playing chess makes kids smarter”; and so on. In academic literature, there are numerous studies investigating the preconditions for learning this game and the effects produced by playing chess on diverse children’ and adults’ development domains.

Chess is considered a sport characterized by high psychophysiological demands, because it requires long training durations, games, and tournaments (Fuentes-García et al., 2020). These could lead to mental, emotional, and physical stress, therefore, it is not a game for everyone. To answer the question what personality characteristics have children who decide to take up chess as a hobby, Bilalić et al. (2007a) have applied the Big Five Questionnaire for Children on 269 primary school children, and they discovered that” children who are less sensitive toward others, more prone to arguing and less to avoiding conflicts (Agreeableness), more energetic (Energy/extraversion), and more open to new experience (Intellect/openness) are more likely to be attracted to the game of chess” (p. 908). Personality traits seems to play an important role only in choosing the chess environment, and not in determining the extent of chess skill development.

Adult chess players have developed some specific features, such as unconventional thinking and orderliness, being more introverted, more intuitive, highly competitive, and more suspicious (Kelly, 1985; Avni et al., 1987) than the general population.

Numerous benefits of playing chess have been researched over time. Several studies are demonstrating the importance of playing chess in developing different skills. In the next section, we will synthetize these findings, accordingly with the previous presented list of child development areas (physical, perceptual and cognitive, language and communication, social, emotional, moral development).

In quotidian common knowledge, chess is considered to be a non-active sport, which needs no physical activities or training, and static chess training is associated with sedentary lifestyle. Although chess does not play a role in the physical development of children, nevertheless a connection between physical exercises and chess can be observed. More precisely, there are studies that investigate the influence that physical condition of chess players has on the performance obtained in competitions. Fornal-Urban et al. (2009) observed that being physically fit is good for chess performance and that the young chess players taking part in their study showed higher level of physical fitness in comparison to their peers from population. They suggest that in the training process of the chess players, more attention should be paid to their fitness preparation, and the tournaments organizers should provide facilities for diverse active forms of recreation. Chess skills alone are not sufficient for performing in chess tournaments, they need to be completed with an adequate physical fitness, in order to endure exhausting chess plays (Golf, 2015; Parra et al., 2020). Thus, we can conclude that combining physical and cognitive training of the chess players might result in a mutual enhancement of both aspects.

A great amount of the perceived benefits of chess underlines cognitive benefits for younger children (Mefoh Philip and Ugwu Lawrence, 2014). For example, Sagar (2020), on the blog named Mind Mentorz, has synthetized the following benefits of learning or playing chess: focus (observation and concentration), analytical observation (finding the pros and cons of various actions), visualization (imagining a sequence of actions before its implementation), foresightedness (aware of or think about the consequences before an action), analytical reasoning (logical vs. impulsive decision making), abstract thinking (consider the bigger picture), planning (develop long term goals and bring them about), and ability to handle multiple considerations simultaneously (weighing various factors at a time). This type of information source can be accessed by parents interested in the game of chess and the development of their children.

Several studies have examined if playing chess exerts a positive influence in developing cognitive abilities and academic performance, to improved mathematical abilities, to solve problems. For example, in an experimental study, Kazemi et al. (2012) demonstrated the correlation between chess and mathematical ability, in primary-and secondary-level male students in Iran. Their results underline that those who played chess scored significantly higher on their meta-cognitive abilities and showed higher problem-solving skills in math. Tachie and Ramathe (2022) conducted a study which concludes that chess training improves both teachers’ and learners’ application of metacognition in supporting learners’ performance in mathematics. Blanch (2022) suggests that schoolchildren who learn or play chess appear to experience a better development of cognitive abilities or bearing a larger improvement in academic performance than schoolchildren who do not learn or play chess.

Counterintuitive findings that there is no evidence that teaching children chess improved their math ability are underlined in other studies. Sala and Gobet (2017) suggest that the effects (if any) of chess instruction, when rigorously tested, appear to be minimal on mathematical abilities and certainly too limited to provide any educational advantage over the traditional instructional methods. Also, no differences were found with respect to metacognitive ability. Another study indicated no correlation between chess playing and mathematical abilities, nor within reading or science capabilities of the students (Jerrim et al., 2018). Their main criticisms regarding the studies which found positive effects of chess on cognitive outcomes consists in the idea that these empirical investigations have not employed rigorous methods in terms of their research design and statistical analyses.

The connection between intelligence and chess was analyzed by Bilalić et al. (2007b) from another perspective, examining the answer to the question whether chess requires intelligence for learning to play it. They have tested children who had recently started to play chess to see how intelligence and practice interact at the beginning of the chess skill acquisition process. Their conclusion is that the role of intelligence in the acquisition of chess skill should not be assessed separately from other relevant factors (e.g., practice, experience, age, and gender). To what extent intelligence, practice, and motivation contribute to chess performance in young children who had just started playing chess was also examined in the study of De Bruin et al. (2014). They discovered that IQ and practice independently predicted chess performance on a chess test at the end of the course. Motivation influenced performance indirectly, by moderating the amount of practice that was undertaken. They concluded that, at the early stages of expertise development, IQ, and motivation influence chess performance.

The influence of learning and practicing chess on executive functions in childhood was the topic of interest in the research designed by Grau-Pérez and Moreira (2017). Executive functions were defined as those cognitive activities that are fundamental to goal-directed behavior and appropriate social conduct, such as: decision making, concept formation, abstract reasoning, working memory, processing speed, interference control, inhibition of impulses, planning, evaluation of errors, cognitive flexibility, shifting focus, organizing, attentional and behavioral control, having access to a complex environment that includes appropriate learning materials, and stimulation in the form of experiences. They discovered that highly complex games like chess can favor the development of planning and flexibility (two executive functions) in childhood.

Waters et al. (2002) have investigated whether one component of intelligence (visual memory ability) was associated with chess skill in a sample of adult chess players. Their data show that visual memory ability did not correlate with chess skill.

Chess can also trigger the creative function in our brain. This game is a challenge to the brain and stimulates both the left and the right brain hemispheres, developing both logical and creative thinking and originality. Sigirtmac (2016) conducted a study to investigate whether chess training has any impacts on creativity and theory of mind skills of children and the results show that the scores of children who had chess training were found to be higher than scores of other children both in creative thinking and theory of mind tests, and the difference between scores of these children were also found to be statistically significant.

Benefits of playing chess have been proven in diverse dimensions of children’s development. One particular are is the development of linguistic intelligence. Joseph et al. (2021) aimed to find an answer to the question: does playing chess improve reading, writing, speaking, and listening skill? And their results indicate significant improvements in verbal reasoning, which is a component of linguistic intelligence.

Gao et al. (2021) have analyzed the link between chess skills and academic performance in primary school students and ascertained that the impact of chess skill on language and mathematics achievement appears to be actually mediated by fluid intelligence. As a feature of cognitive functioning, fluid intelligence is the ability to think speedily about new situations, to solve unfamiliar problems by using critical thinking, flexibly, and logical reasoning.

The literature on benefits of playing chess has provided some useful insights into understanding the emotional development of players. Chess players have differentiated from the general population through higher emotional stability and the ability to manage emotions (Avni et al., 1987). The same idea is confirmed by Aciego et al. (2012), who found that chess improves, besides cognitive abilities, coping and problem-solving capacity, also socio-affective development of children and adolescents who practice it.

Chess training as an intervention method, used by Jamshidi (2021) in her semi-experimental research in Iran, has improved emotional intelligence and its components in fourth to sixth grade of elementary school female students with math disorders. The results showed that chess also improves trust in problem solving and personal control in the participants.

Likewise, chess training program might reduce the level of risk aversion. Islam et al. (2021) has found that children in primary school who were taught the game of chess, and played regularly, were more likely to be less risk averse than their peers. These results are explained in connection with the exposure to win/loss situations and competition, as well as with teaching children the potential benefits of strategic risk-taking. Such programs are an adequate way to help young people deal with risk and reward later in life.

Chess is also associated with emotion regulation, which is a term that consists in the ability to process, influence, and control our emotional state. Blanch and Llaveria (2021) pointed out that chess players scored higher in expressive suppression compared with the general population. Expressive suppression is a form of emotion regulation that involves conscious, voluntary inhibition of the outward manifestation of an ongoing emotional response (Gross, 2013).

Aside from developing cognitive skills, chess also develops children’s social skills. A benefit for children of playing games with rules consists in developing social aspects, such as taking turns, learning fair play, self-respect and respect for others, understanding others’ perspectives, and developing empathy. Joseph et al. (2016) underline the idea that chess might serves as a bridge in educational contexts, bringing together children of different ages, races, and genders in an activity they can all enjoy. Chess might help build individual friendships and team cohesion when children compete against other schools.

Negative aspects can also be observed in the dynamics within chess communities. For example, Puddephatt (2008) identifies some organizational mechanisms that operate in the world of chess, such as: isolation from competing social spheres; encapsulation within a symbolic status structure; a collective feeling of elite status; trials of worthiness; and prestructured ritual.

According to Mathieson (2003), moral maturity consists of seven elements: moral agency, harnessing cognitive ability, harnessing emotional resources, using social skill, using principles, respecting others, and developing a sense of meaning.

Chess players learn early that every move and decision comes with a consequence. Tanajyan et al. (2021) show, in their study, that chess promotes the ability to foresee, influencing the situation.

Dealing with adverse circumstances over time, chess players develop their maturity, patience, and composure. In their innovative study where students engaged in self-directed activity goals during their holiday, Tam et al. (2023) found that students who played chess experienced improved attitudes toward homework and perceived that learning chess resulted in improvements within their observation and patience skills.

These skills that are developed by playing chess could provide chess players a certain advantage in facing quotidian challenges.

In the literature, the studies are, rather, oriented toward the discovery of the advantages of playing chess. The negative impact on the players, on their social dynamics is less explored. Our study aims to present the parents’ perception of chess as balanced as possible, investigating both the benefits and the disadvantages perceived by them.

## 2. Materials and methods

### 2.1. Research questions

In this paper, we explore parents’ beliefs about the importance of playing chess in children’s development. The study was conducted in Romania, and the motivation for conducting such a study is represented by the fact that research on this matter is poor in Romania and by the fact that chess has gained importance over the years and the interest for analyzing its effects on the development of children increased worldwide.

RQ1: What are the parents’ perceptions about chess’s role in their children’s development?

RQ2: How does parents’ perception differ depending on whether or not they know how to play chess?

RQ3: What is the profile of the children’s parents who play chess?

### 2.2. Data collection method

We conducted an online survey to determine parents’ perceptions of the advantages and disadvantages for their children to play chess. Data were collected through an online survey through the free application Google forms in 2021. The population of the study is represented by parents of chess-playing children who are members of chess clubs from Romania (from all the counties). It was selected in a non-probabilistic way, and the questionnaire link was sent by email to all members of chess clubs, and 774 responses were collected. The survey took approximately 30 min. The research received the approval of the Ethics Commission in social research from Transilvania University of Brasov, Romania.

### 2.3. Sample

The questionnaire was applied to 774 respondents. The respondents have an average of 42 years (M = 41.8, SD = 7.1), and the majority of respondents are from the urban area, (87.3%), have tertiary education level (58.6%) and they know how to play chess (66.7%). Most of them have a medium-high level of income. Only a third of them are members of chess clubs (27.9%). The characteristics of the respondents are presented in Table 1.

**Table 1:** Sociodemographic characteristic of respondents (*N* = 774).

	Category	Count	Percentage
Residential environment	Rural	98	12.7%
	Urban	676	87.3%
Highest academic degree	Primary education	2	0.3%
	Secondary education	99	12.8%
	Tertiary education	451	58.3%
	Postgraduate education	218	28.2%
	NA	4	0.5
The income level-self-perception	Very low	3	0.4%
	Low	33	4.3%
	Medium	492	63.6%
	High	220	28.4%
	Very high	14	1.8%
	NA	12	1.6%
Playing chess	Yes	516	66.7%
	No	258	33.3%
Member in chess club (during the life)	Yes	216	27.9%
	No	558	72.1%

### 2.4. The research instrument

A non-standardized questionnaire was used with items related to the three research questions. The questionnaire was built by the authors of this paper and it was presented to the parents in Romanian. In the present study, only a part of the data is presented. For the first research question, the following items were included: q1: The first word associated with the term chess (open-ended); q9, q10: the degree to which the chess has an impact on the players (positive and negative aspects that are presented in Table 2; 4 = very high degree, 1-very low degree); q18: the degree to which chess can help children to pass over the negative emotions (5 = very high degree, 1-very low degree); q19: types of negative emotions that can be overcome through chess (the fear of the unknown, the fear of expressing oneself, the impatience); q20: the degree to which chess could help children to develop positive emotions (5 = very high degree, 1-very low degree); q21: if the children made new friends in chess club (yes, no); q27: the degree to which the children who play chess are different from those who do not do this (5 = very high degree, 1-very low degree); q28: the words that describe best the children who play chess (open-ended); and q41: Preconceptions regarding children who play chess (disadvantages; open-ended). The research instrument can be found in Appendix 1.

**Table 2:** Positive and negative impact on the chess–perceptions of the parents.

Positive aspects (q9; 4 = very high degree, 1 = very low degree)	*M*	SD	Negative aspects (q10; 4 = very high degree, 1 = very low degree)	*M*	SD
School performance	3.35	0.680	Withdrawal and decreased sociability	1.37	0.584
Competitive spirit	3.48	0.657	Decreased mental flexibility (difficulty thinking outside the box)	1.23	0.472
Spirit of cooperation	3.08	0.775	Decreased self-esteem as a result of setting negatives that are too high	1.40	0.582
Communication skills	3.02	0.779	Deprivation of more fun age-appropriate activities	1.79	0.777
Relationship skills	3.31	0.705	Atrophy of emotional capacities	1.20	0.459
Mental abilities	3.82	0.401	Atrophy of other senses to the negative of the visual one	1.19	0.448
Emotional capacities	3.39	0.652	Disinterest in other areas of life	1.43	0.620
Strong character	3.42	0.654	Infatuation and selfishness	1.36	0.621
Self-discipline	3.35	0.722	Mental fatigue as a result of excessive egati exposure	1.82	0.864
Ability to make quick decisions	3.25	0.825	Addiction to adrenaline and competition	1.36	0.644
High self-esteem	3.27	0.738	Robotization in social interactions	1.19	0.438
Intuition	3.38	0.747	Negative impact on mental health	1.10	0.370
			Increasing aggression	1.14	0.409
Index	3.34	0.40	Index	1.36	0.38

For the second research question, all the results regarding research question 1 were compared to two categories of parents: those who know to play chess and those who do not know.

For the third research question, the following items were used: q8: The reasons the children take chess lessons (open-ended); q23: If the parents made friends at the chess clubs (yes/no); q25: If they feel that they are part of a community among other parents (yes, no); q29: the degree to which they feel they are different from other parents who have children who do not play chess (5 = very high degree, 1 = very low degree); q30: Describing the parents who have children at chess classes (open-ended); q44: The degree to which the competitions are essential for them (5 = very high degree, 1 = very low degree); q51: self-perception of the parent’s income level, who have children that play chess (5 = very high income, 1 = very low income); and q52: self-perception of the parent’s education, who have children that play chess (intellectual, workers, and both categories) and also sociodemographic items (Table 1). Furthermore, considering the validity of the research, the authors took into account the theoretical information from the literature regarding the development of a questionnaire. The authors of the paper configured the dimensions, and operationalized the concepts in accordance with the theoretical approaches identified at the current stage of the research. Moreover, the questionnaire was pre-tested before disseminating it, in order to guarantee the validity of the instrument. Thus, the questionnaire was applied to 50 respondents in the pre-testing stage.

### 2.5. Data analysis

The data were analyzed using IBM SPSS Statistics (version 23). Answers to open-ended questions were analyzed using content analysis. The answers were coded and merged into some categories presented in Appendix 1. For close-ended questions were used descriptive statistics (percentages, mean, and standard deviation). Comparisons were made depending on whether parents knew or not to play chess with the Independent Samples *t*-Test. For the variables, q9: positive aspects and q10: negative aspects that were complexly measured through several indicators, indexes Mesesan-Schmitz and Coman (2020) were made to measure the information synthetically.

## 3. Results

### 3.1. RQ1: what are the parents’ perceptions about chess’s role in their children’s development?

Parents consider there are many benefits of playing chess. At question q1, the first word associated with the term chess, most of the respondents associate chess with cognitive abilities (55.8%) as cognitive skills (e.g., mathematical skills, analysis and strategy, anticipation, training the mind, attention, self-control, mental calculation, concentration, neural connections, creativity, complexity, intuitive thinking, intelligence/high IQ, and memory). Those advantages are the main reason why parents want their children to take chess courses (Appendix 2, Table 1A). Parents believe that their children (who play chess) are talented people 4.5% and special people 3.6%, more intelligent and smarter than others (24.4%), capable of paying attention and concentrating (6.2%), persevering people 3.6%, motivated to study, to learn new things 2.6%, good at logical games/logical skills 3.1% (Appendix 2, Table 1B). It is observed that parents pay attention to cognitive abilities. 46% declared their children are different from those who do not play chess through cognitive abilities and 31% through character traits.

These results are also confirmed by the answers for q9. At this question, the parents gave the highest score to the development of cognitive abilities (M = 3.82, SD = 4.01) as a positive aspect. In addition, they also mentioned character traits such as strong character (M = 3.42, SD = 0.65), and competitive spirit (M = 3.48, SD = 0.65).

Analyzing the answers of the q10 (Table 2), results that the parent does not perceive any risk for their children (M = 1.36, SD = 0.38). However, they only see positive aspects (M = 3.31, SD = 0.8). Only a small part of them considers that the children can sometimes suffer from the deprivation of more fun, age-appropriate activities (M = 1.79, SD = 0.77) or mental fatigue due to exposure to excessive stress (M = 1.82, SD = 0.86). At the open-ended q41, parents declared that there is some possible risk for the children, seen more as preconceptions of others (Appendix 2, Table 1C). The persons who play chess do not do sport and are predisposed to sedentarism (5%). They do not develop social abilities (7.4%) and develop arrogance and individualism. Their social life is placed in a closed circle. Chess is the sport of misfits, antisocials, “nerds,” and weird/individualists/autists. They need to spend much free time preparing for competitions and do not have sufficient time for homework. In addition, chess players have no prospects after junior ageand they do not receive sufficient financial incentives. There is the risk that the children do only this (play chess) and nothing else age specific. All these results are presented in Appendix 1. Even if the parent did not associate chess with social abilities, the results of question q21, 84.6%, answered that the children succeeded in making new friends (Table 3). Also, parents perceived that chess could help their children develop positive emotions (M = 4.38, SD = 0.78) and overcome negative emotions (M = 4.20, SD = 0.84), especially impatience (41.6%; Table 3).

**Table 3:** The impact of the chess—perceptions of the parents.

Positive aspects (5 = very high degree, 1 = very low degree)	Negative aspects (5 = very high degree, 1 = very low degree)
Positive aspects index	Negative aspects index
M = 3.34, SD = 0.40	M = 1.36, SD = 0.38
Q20: Chess helps the children to develop positive emotions M = 4.38, SD = 0.78	Q18: Chess helps the children to overcome the negative emotions M = 4.20, SD = 0.84
	The fear of the unknown 27.3%
	The fear of expressing oneself 25.6%
	The impatience 41.6%
	Another 5.6%
Q1. The chess is associated with the development of:	Q41: Preconceptions
55.8% cognitive abilities	The chess community is not very visible/it is not visible in the mass media/it cannot be watched on TV because it takes too long 11.9%
	Reduced physical abilities/predisposition to sedentarism 5.0%
	Financial rewards reduced/not commensurate with investments 3.5%
	Arrogance 2.3%
	That it is the sport of misfits, antisocials, “nerds,” and weird/individualists/autists 7.4%
	That they are introverts 2.2%
	Poor communication 1.7%
	Individualist 1.9%
	Lack of money 3.1%
	Lack of horizons after finishing junior studies 1.3%
	Takes up too much free time for preparation 1.6%
Q28: The children are different from those who do not play chess	
Cognitive abilities	
Good at logic games/logical skills 3.1%	
Able to pay attention/concentrate 6.2%	
Smarter/intelligent than the rest	
24.4%	
Motivated to study, to learn new things 2.6%	
Character traits	
Ambitious 2.6%	
Curious 2.2%	
Responsible 2.3%	
Persevering 3.6%	

### 3.2. RQ2: how does parents’ perception differ depending on whether or not they know how to play chess?

There are some differences in perception between parents who know whether to play chess or not. The parents who know chess give higher scores to the impact of chess on positive aspects [Appendix 2, Table 2A; M1 = 3.37, SD1 = 0.39; M2 = 3.20, SD2 = 0.38; *t*(773) = 5.79. *p* < 0.001], especially when it comes to the competitive spirit, spirit of cooperation, mental abilities, emotional capacities, strong character, self-discipline, ability to make quick decisions, high self-esteem, and intuition. The same parents consider in higher degree that chess helps the children to develop positive emotions [q20; *t*(772) = 3.63; *p* < 0.001] and helps the children to overcome the negative emotions (q18), [*t*(772) = 2.96; *p* < 0.001; M1 = 4.24, SD1 = 0.80; M2 = 4.38, SD2 = 0.78]. There are no differences regarding negative aspects {M1 = 1.30, SD1 = 0.41; M2 = 1.34, SD2 = 0.31; [*t*(651) = 0.93. *p* < 0.001]}. Those who know how to play chess consider to a higher degree that children can develop an addiction to adrenaline from competition (M1 = 1.41, SD1 = 0.67; M2 = 1.27, SD2 = 0.56) or that chess can have a negative impact on mental health (M1 = 1.12, SD1 = 0.41; M2 = 1.05, SD2 = 0.24). Moreover, our results also showed that compared to parents who do not know how to play chess, parents who know how to play chess believe to a greater extent that chess influences and promotes the development of strong character in children [q9; *t*(761) = 5.117, *p* < 0.05; M1 = 3.50, SD1 = 0.62; M2 = 3.25, SD2 = 0.68; Table 4].

**Table 4:** Independent sample *t-*test for the role of chess in developing strong character and parent’s knowledge of playing chess.

	*t*-test for equality of means										
	Group	*N*	Mean	S. D.	*t*	df	*p*	Mean difference	Std. Error difference	CI4	
	Do you know to play chess									Lower	Upper
Q9: To what extent do you think that chess can contribute to the development of strong character in children?	Yes	509	3.50	0.62	5.117	761	0.000	0.25	0.04	0.15	0.35
	No	254	3.25	0.68							
										
										

Furthermore, our results also showed that parents who play chess are more satisfied with the amount of knowledge their children have acquired during the courses, than parents who do not know how to play chess. In other words, the average of people who know how to play and are satisfied with the knowledge their children have from chess lessons is higher than the average of people who do not know how to play chess [q11; *t*(772) = 2.096, *p* < 0.05; M1 = 4.07, SD1 = 0.85; M2 = 3.93, SD2 = 0.79; Table 5].

**Table 5:** Independent sample *t*-test for parents’ satisfaction regarding knowledge acquired by children and parents knowledge of playing chess.

	*t*-test for equality of means										
	Group	*N*	Mean	S. D.	*t*	df	*p*	Mean difference	Std. Error difference	CI4	
	Do you know to play chess									Lower	Upper
Q11: To what extent are you satisfied with the amount of knowledge the child has acquired during chess training? 1 = to a very small extent; 5 = to a great extent	Yes	516	4.07	0.85	2.096	772	0.036	0.13	0.06	0.00	0.25
	No	258	3.93	0.79							
										
										

### 3.3. RQ3: what is the profile of the children’s parents who play chess?

Parents are persons with a medium-high income with at least a bachelor’s degree. Most of them knew to play chess, and a third was chess club members. They made friends with other club members and interacted with them. There are people for whom competition and the development of cognitive abilities are the primary concerns. Their self-perception is that they are somehow different, they are more than others involved in children’s education (11.9%), pay attention to the teaching (4.4%), and they are open minded (3.6%), Willing to offer in advance and get involved in the children’s activity, financially (7.2%), breathe (7.1%), intelligent (4.7%), ordinary (3.6%), and responsible 11.5% (Appendix 2, Table 1D; Table 6).

**Table 6:** The profile of chess playing children’s parents.

Q23: 70.9% made friend in chess clubs	Q44: They feel that competition is very important for them
Q8: 40.7% decided their children take chess classes because this activity is utile for children’s development	(5 = very high degree, 1-very low degree)
	M = 3.79, SD = 0.99
Q25: They feel that together with the others parent belong to the community of chess club	Q51: 78.8% of parent consider that they have an income medium to high level
(5 = very high degree, 1-very low degree)	
M = 3.53, SD = 1.29	
Q29: They feel that the parents of the children who take chess courses are different	Q52: 53.1% of parents consider that the parent are intellectual and 46.6% intellectuals + workers
(5 = very high degree, 1-very low degree)	
M = 3.38, SD = 1.02	
Q30: The parents of the children who take chess classes are	
Involved in children’s education (11.9%)	
Pay attention to the education (4.4%)	
They are open minded(3.6%)	
Willing to offer in advance and get involved in the children’s activity, financially (7.2%)	
Breathe (7.1%)	
Intelligent (4.7%)	
Ordinary (3.6%)	
Responsible 11.5%	
etc.	

## 4. Discussion and conclusion

In this study, we analyzed Romanian parents’ perception about the role of chess in the development of their children. The study was conducted in Romania, and during our research we were interested in finding out the parents’ perspectives about the way chess influences the development of their children, in finding out if there are any differences in the parents’ opinions depending on whether they know how to play chess or not, and we were interesting in gathering information about the profile of the children’s parents who play chess.

The results of our research showed that parents are of the opinion that chess helps children develop their cognitive abilities, their character and their competitive spirit. From this point of view, our research is in line with a previous study (Sagar, 2020), which showed the cognitive benefits that chess has on children. Most of the parents focused on highlighting only the positive influence of chess on the development of their children, and those who also mentioned the negatives aspects of playing chess referred to the idea that children who play chess could be deprived of other fun activities, or that they could experience fatigue due to the stress created by the game.

In the context of the positive effects of chess, our research is in line with a previous study (Aciego et al., 2012) which highlighted the positive effects of chess on the socio-affective development of children and adolescents. In this regard, our results showed that, parents considered that chess helped their children develop positive emotions and helped them overcome negative emotions by teaching them how to become patient. Furthermore, when discussing the perceptions of parents about the effects of playing chess, an important matter that should be taken into consideration is the matter of parental support. Thus, parents of children who play chess should receive support in order to sustain their children in the process of playing chess (Moè et al., 2020).

However, the results of the study revealed that parents perceived that children who play chess could be at risk due to the stereotypes that exist at societal level about chess players. The stereotypes mentioned most often by the parents referred to the fact that chess players do not do sports, that they do not have social abilities and might be perceived as arrogant people, or that children spend too much time playing chess and as a result, they do not have time to carry on other types of activities.

Considering the opinions of parents who play or who do not play chess, our results revealed differences between the opinions of the two categories of parents. From this perspective, our research is in line with a previous study (Horgan, 1992), which highlighted the fact that people who do not play chess can sometimes have wrong opinions about the game and its effects on children. Thus, considering parents’ opinions about their children’s chess performance, Horgan (1992) demonstrates that parents who are not chess players have misperceptions about their children’s performance. The parents whose children have lower ratings stated that they have worse results in the first rounds because they are more stressed at the beginning of the championships, and the parents whose children have better ratings stated that the reasons why their children underperform in the final rounds is represented by the fact that they are becoming tired or they lose focus. The researcher mentions that these wrong perceptions of parents are due to their lack of knowledge about chess and about the system according to which the players are assigned.

Given the results of our research, parents who do know how to play chess were more likely to focus on the positive effects of the game on the development of their children. For example, in the context of the development of strong character through chess, parents who know how to play chess and have more knowledge about the game, recognize to a greater extent than parents who do not know how to play chess, the positive influence of chess on the formation of strong character. A possible explanation for this difference of opinion may be that parents who know how to play chess have personally experienced the benefits of this sport and know better how it can contribute to strengthening the character of individuals. Moreover, according to the results, parents who know how to play chess are more satisfied with their children’s accumulated knowledge following chess lessons. This may be due to the fact that parents who know the rules of the game may recognize their children’s progress and development more easily and quickly than parents who do not know what the game entails.

In the context of the negative effects of chess, parents who do know how to play, were also able to recognize some of these negative effects, them mentioning that it is possible for children to become addicted the adrenaline felt during the game, and that chess could sometimes negatively impact the mental health of their children.

Given the profile of the parents of children who play chess, our study showed that these parents have high educational levels (at least a bachelor’s degree), that they are mostly people who know how to play chess, who were members of a chess club and who made friends with other parents from the chess club. The parents who participated in the study are people who put great value on the development of cognitive abilities and who are in favor of competition. They think of themselves as people who are somewhat more concerned with the development of their children and with the education of their children, they see themselves as open minded people, who invest money in the education of their children.

According to the results of the conducted research, the general opinion of the parents about the role of chess in children’s development is a positive one. Similar to the results of other studies (Horgan, 1987), this research supports the idea that playing chess positively influences the development of children’s mental capacity. Furthermore, by acknowledging the benefits of playing chess and by analyzing the effect of chess on the development of children, previous studies postulated the idea that that chess could be introduced in the school curriculum (Gliga and Flesner, 2014; Jankovic and Novak, 2019). Furthermore, Jankovic and Novak (2019) support its introduction into the school curriculum and emphasize the fact that one of the reasons why chess is not integrated into the educational process is due to the lack of knowledge about the multitude of benefits that chess has on children. Taking this aspect into account, through our research, we have highlighted the benefits and advantages of playing chess in the opinion of parents whose children play chess, and thus, the paper can contribute to raising awareness regarding the positive effects of playing chess.

### 4.1. Limitations and future research directions

Although our research provides relevant and important information regarding the benefits of chess on children’s intellectual and social development, it also has certain limitations. One of the limitations of the research is shown by the fact that the participants of the study were only represented by parents, and in the future the opinion of the children who play chess, about its negative or positive effects, should also be analyzed. In this regard, the validity of the data could be tested in a future research by measuring children’s opinions about chess, in order to compare their answers with the answers of the parents. Another limitation is represented by the fact that the study was conducted only in Romania, and thus its results are relevant in the Romanian context, but in order to confirm (or not) the results of our study, in a future research the opinion of parents from other countries should also be taken into consideration. Furthermore, the questionnaire was applied only to parents whose children play chess or take chess lessons, and in the future the opinion of parents whose children do not play chess should also be measured to see how they perceive the benefits of chess and how they would relate to the possibility of integrating chess as a subject in the school curriculum. Moreover, a future research could also focus on examining the opinion of other people who interact with children who play chess (e.g., teachers), about the role of chess in the development of children, and the research could focus more on the negative aspects, which are less examined in the literature. Even more, a future research should also take into consideration the matter of parental support and should focus on the process of adopting supportive practices for parents in order to favor chess play in children and adolescents.

## Data availability statement

The original contributions presented in the study are included in the article/Supplementary material; further inquiries can be directed to the corresponding author.

## Ethics statement

The studies involving human participants were reviewed and approved by Research Ethics Commission of Transilvania University of Brasov. The patients/participants provided their written informed consent to participate in this study.

## Author contributions

CN, CC, and MB contributed to the conception and design of the study. LM-S, MG, and IA organized the database. LM-S and CN performed the statistical analysis. CN and CC wrote the first draft of the manuscript. CN, CC, MB, LM-S, MG, IA, IT, and IN wrote the sections of the manuscript. All authors contributed to the article and approved the submitted version.

## Conflict of interest

The authors declare that the research was conducted in the absence of any commercial or financial relationships that could be construed as a potential conflict of interest.

## Publisher’s note

All claims expressed in this article are solely those of the authors and do not necessarily represent those of their affiliated organizations, or those of the publisher, the editors and the reviewers. Any product that may be evaluated in this article, or claim that may be made by its manufacturer, is not guaranteed or endorsed by the publisher.
