# Supplementary material for: The role of chess in the development of children-parents’ perspectives
Source: Front Psychol. 2023 Jun 26;14:1210917. doi: 10.3389/fpsyg.2023.1210917 (PMC10330813; doi:10.3389/fpsyg.2023.1210917)
Supplement: Supplementary file 1 [file Data_Sheet_1.doc]

1. **Appendix 1. Questionnaire**

1. What is the first word that comes to your mind when you hear CHESS? ..............................

2. What image do you think chess has in Romania?

• A very good picture

• A good image

• A neither good nor bad image

• A bad image

• A very bad picture

3. Do you know how to play chess?

•Yes

•Not

4. Have you ever participated in a chess club?

•Yes

•Not

5. (if yes), How long have you been part of such a club?

................................years

6. How did you find out about the children's chess courses? (ONE ANSWER)

•From the Internet

• From the television

• From the print media

• From friends, acquaintances

• From school or kindergarten

• Other (note which ones?)……………………………….

7. What motivated you more to enroll your child(ren) in chess classes? (ONE ANSWER)

• The child's wish

• My wish (or my partner's)

• Desire of other family members (parents, in-laws, other relatives)

• Desire of other people (specify which people) ....................

8. What did you have in mind when you decided to enroll your child in chess? (ONE ANSWER)

• The desire to involve him in a useful activity

• The desire to involve him in an enjoyable activity

• The desire to involve him in an activity, whatever it may be

• The desire to involve him in an activity ………………............

9. I am going to present you with a series of positive characteristics, asking you to tick to what extent you think that playing chess can contribute to their development in children:

|  | To a very large extent | To a large extent | To a small extent | To a very small extent |
| --- | --- | --- | --- | --- |
| School performance |  |  |  |  |
| Competitive spirit |  |  |  |  |
| Spirit of cooperation |  |  |  |  |
| Communication skills |  |  |  |  |
| Relationship skills |  |  |  |  |
| Mental abilities |  |  |  |  |
| Emotional capacities |  |  |  |  |
| Strong character |  |  |  |  |
| Self-discipline |  |  |  |  |
| Ability to make quick decisions |  |  |  |  |
| Social integration |  |  |  |  |
| High self-esteem |  |  |  |  |
| Intuition |  |  |  |  |
| Other positive characteristic (specify which) ............ |  |  |  |  |

10. I am going to present you with a series of negative aspects, asking you to tick to what extent you consider that playing chess can contribute to their manifestation in children:

|  | To a very large extent | To a large extent | To a small extent | To a very small extent |
| --- | --- | --- | --- | --- |
| Withdrawal and decreased sociability |  |  |  |  |
| Decreased mental flexibility (difficulty thinking outside the box) |  |  |  |  |
| Decreased self-esteem as a result of setting standards that are too high |  |  |  |  |
| Deprivation of more fun age-appropriate activities |  |  |  |  |
| Atrophy of emotional capacities |  |  |  |  |
| Atrophy of other senses to the detriment of the visual one |  |  |  |  |
| Disinterest in other areas of life |  |  |  |  |
| Infatuation and selfishness |  |  |  |  |
| Mental fatigue as a result of exposure to excessive stress |  |  |  |  |
| Addiction to the adrenaline rush of competition |  |  |  |  |
| Robotization in social interactions |  |  |  |  |
| Negative impact on mental health |  |  |  |  |
| Increasing aggression |  |  |  |  |
| Other negative aspect (specify which) ......................... |  |  |  |  |
| None |  |  |  |  |

11. To what extent are you satisfied with the amount of knowledge the child has acquired during chess training?

• To a very large extent

• Largely

• So and so

• To a small extent

• To a very small extent

12. To what extent are you satisfied with your child's performance as a result of chess training?

• To a very large extent

•Largely

•So and so

• To a small extent

• To a very small extent

13.To what extent would you recommend your friends enroll their children in chess clubs?

• To a very large extent

•Largely

•So and so

• To a small extent

• To a very small extent

14. What would be the main argument you would bring to a friend to convince him to enroll his child in chess?

................................................ ................................................ ..

15. To what extent do you consider that the environment within the club is suitable for your child?

• To a very large extent

•Largely

•So and so

• To a small extent

• To a very small extent

16. How would you describe this entourage in one word?

................................................ ..

17. In your opinion, what type of values and character traits predominantly form the practice of chess? (ONE ANSWER)

• None

• Self-discipline

• Self-control

• Tolerance

•Patience

• Respect for authority

•Self respect

• Responsibility

•Correctness

• Perseverance

•Team spirit

• Competitiveness

• Orientation towards success

• Empathy

• Friendship

• Individualism

• Other (write down which) ............................................ .................

18. To what extent do you consider that chess lessons can help the child overcome certain negative emotions (fear of the unknown, fear of expressing himself, restlessness, impatience)?

• To a very large extent

•Largely

•So and so

• To a small extent

• To a very small extent

19. Which negative emotion do you think can be overcome the most as a result of playing chess? (ONE ANSWER)

• Fear of the unknown

• Fear of expressing oneself

• Restlessness

• Impatience

• Other (write down which one)................................................. ........................

20. To what extent do you think that chess lessons can help the child develop certain positive emotions?

• To a very large extent

•Largely

•So and so

• To a small extent

• To a very small extent

21. Has your child made friends in the chess class that he also meets in his free time?

•Yes

•Not

•I do not know

22.(if yes) About how many friends did he make? ..................

23. You have you made friends among the parents of the children who participate in the chess club with whom you also meet in your free time?

•Yes

•Not

24.(if yes) About how many friends have you made? ...................

25. To what extent do you feel that you form a community together with the parents of the children at the chess club?

• To a very large extent

•Largely

•So and so

• To a small extent

• To a very small extent

26. How would you characterize the community/group of parents and children participating in the chess club in one word? ............

27. To what extent do you think that those children who play chess are different from those who do not?

• To a very large extent

•Largely

•So and so

• To a small extent

• To a very small extent

28. How would you describe the children who play chess in one word? ................................................ .........

29. To what extent do you think that parents of children who decide to send their children to play chess are different from parents who send their children to practice other activities?

• To a very large extent

•Largely

•So and so

• To a small extent

• To a very small extent

30. How would you describe in one word the parents who decide to send their children to play chess? ..

31. How would you characterize the community of chess practitioners in one word? ..........

32. Before coming to the club, to what extent did you anticipate your children's attraction to this sport?

• To a very large extent

•Largely

•So and so

• To a small extent

• To a very small extent

33. What do you consider to be the greatest benefit of the child's participation in chess competitions? (ONE ANSWER)

• Accumulation of experience

• Socializing with other children from other cultures

• Strengthening character

• Overcoming emotional barriers

• Experiencing intense emotional experiences

• Increasing self-esteem

• The pride of representing your country internationally

34. To what extent do you think that chess is appreciated rather in certain countries than others?

• To a very large extent

•Largely

•So and so

• To a small extent

• To a very small extent

35. In which countries do you think chess has caught on better?………………...

36. In which countries do you think chess has not caught on?…………………….....

37. To what do you think these differences between countries in terms of interest in chess are due?................................. .............

38. What image do you think chess has in Romania?

• A very good image

• A good image

• An image neither good nor bad

• A bad image

• A very bad image

39. But chess players, what image do you think they have?

• A very good image

• A good image

• A neither good nor bad image

• A bad image

• A very bad image

40. To what extent have you personally encountered people who are prejudiced about chess and chess players?

• To a very large extent

•Largely

•So and so

• To a small extent

• To a very small extent

41.In your opinion, what would be the main stereotype about chess and chess players?

…………………………………………………………………………

42. Briefly describe how you think children who play chess are seen in Romania?

…………………………………………………………………………

43. What aspects about children who play chess are less known to Romanians?

…………………………………………………………………………

44. To what extent is the chess performance important to you?

• To a very large extent

•Largely

•So and so

• To a small extent

• To a very small extent

45. Your age ……………………

46. Your occupation………………………………

47. Environment of origin

• Urban

• Rural

48. In which county were you born? ......................................... ..................

49. Your education

• Primary education (general school, gymnasium)

• Secondary education (vocational school, high school)

• Tertiary studies (faculty with or without master's degree)

•Postgraduate studies

50. From your point of view, how do you assess the level of income in your family?

•Very high

•Picked up

• Environment

• Low

• Very low

51. From what you have seen, parents who decide to send their children to

chess, tend to have an income level rather:

•Very high

•Picked up

• Environment

• Low

• Very low

• All variants above

52. From what you have seen, parents who decide to send their children to

chess, tend to be:

• Intellectuals

• Workers

• Both of the above options

53. How long has your child participated in chess lessons?... (express the answer in years)

54. How old is your child? (express the answer in complete years)

55. In your experience, how do people who play chess differ from those who do not?

..........................................................

**Appendix 2**

**Table 1.a. What do parents associate chess with? (q1)**

| **Dimensions-positive** | **Count** | **Percentage** |
| --- | --- | --- |
| Cognitive abilities  (cognitive skills, mathematical skills, analysis and strategy, anticipation, training the mind, attention, self-control, mental calculation, concentration, neural connections, creativity, complexity, intuitive thinking, intelligence/high IQ, memory) | 432 | 55.8% |
| Character traits  (ambition, perseverance, calmness, competitiveness/fighter character/performance, fairness, personal development, discipline, trust, organization, patience) | 12 | 1.6% |
| Emotions  (pleasure, emotions, peace of mind, optimism, passion, relaxation) | 7 | 0.9% |
| Social abilities  (connections, openness, fun, sometimes moral and material support in life so far) | 6 | 0.8% |
| Way of education | 4 | 0.5% |
| Important Chess players | 12 | 1.6% |
| Championships | 27 | 3.5% |
| Maestro | 5 | 0.6% |
| Pieces of chess | 97 | 12.5% |
| Others | 103 | 13.3% |
| NA | 69 | 8.9% |
| **Total** | **774** | **100%** |

**Table 1.b. Words that describe the best children who play chess-q28-positive aspects**

|  | Count | Percentage |
| --- | --- | --- |
| **Cognitive abilities** | | |
| Skilled/agile | 16 | 2.1% |
| time management skills | 5 | 0.6% |
| math skills/real sciences | 14 | 1.8% |
| analytical | 2 | 0.3% |
| trained to think | 10 | 1.3% |
| self-taught | 1 | 0.1% |
| good at logic games/logical skills | 24 | 3.1% |
| good at school | 1 | 0.1% |
| able to make decisions on their own | 1 | 0.1% |
| ability to anticipate | 1 | 0.1% |
| able to pay attention/concentrate | 48 | 6.2% |
| creative | 9 | 1.2% |
| resourceful | 2 | 0.3% |
| smarter/intelligent than the rest | 189 | 24.4% |
| memory | 1 | 0.1% |
| motivated to study, to learn new things | 20 | 2.6% |
| intuitive | 6 | 0.8% |
| adults | 6 | 0.8% |
| Total | **356** | **46%** |
| **Character traits** | | |
| activity | 2 | 0.3% |
| ambitious | 20 | 2.6% |
| calculate/balance | 13 | 1.7% |
| capable of additional effort/sacrifice/renunciation | 12 | 1.6% |
| strong character | 5 | 0.6% |
| competitive | 15 | 1.9% |
| correction | 1 | 0.1% |
| brave/casual | 9 | 1.2% |
| curious | 17 | 2.2% |
| open | 3 | 0.4% |
| disciplined | 12 | 1.6% |
| enthusiasm | 3 | 0.4% |
| determined/dedicated/ambition | 8 | 1.0% |
| confidence in one's own strength | 9 | 1.2% |
| independent | 5 | 0.6% |
| individualists/isolated | 2 | 0.3% |
| understanding | 2 | 0.3% |
| workers | 12 | 1.6% |
| friendly | 6 | 0.8% |
| reason | 2 | 0.3% |
| stronger | 4 | 0.5% |
| persevering | 28 | 3.6% |
| patient | 12 | 1.6% |
| responsible | 18 | 2.3% |
| ordered/organized | 7 | 0.9% |
| Subway | 4 | 0.5% |
| Perfectionists | 1 | 0.1% |
| Performance | 1 | 0.1% |
| professional | 1 | 0.1% |
| Serious | 5 | 0.6% |
| **Total** | **239** | **31%** |
| Emotions | | |
| happy that they have a passion | 2 | 0.3% |
| worry | 1 | 0.1% |
| emotional intelligence | 3 | 0.4% |
| **Total** | **6** | **0.8%** |
| **Others** | | |
| civilized/educated | 4 | 0.5% |
| children with involved parents | 3 | 0.4% |
| special/special | 28 | 3.6% |
| I am part of an exclusive group | 1 | 0.1% |
| Looking for hobbies | 1 | 0.1% |
| more chances in life | 3 | 0.4% |
| interest | 2 | 0.3% |
| lucky | 3 | 0.4% |
| rule | 11 | 1.4% |
| they are good at everything | 1 | 0.1% |
| talent | 35 | 4.5% |
| other | 40 | 5.2% |
| N/A | 41 | 5.3% |
| Total | 173 | 22.2% |
| **TOTAL** | **774** | **100%** |

**Table 1.c. How chi**ldren playing chess are described by others -the preconceptions q41-negative aspects

|  | Count | Percentage |
| --- | --- | --- |
| **Chess community aspects** |  |  |
| The great effort made by the parents | 2 | 0.3% |
| The chess community is not very visible/it is not visible in the mass media/it cannot be watched on TV because it takes too long | 92 | 11.9% |
| **Chess aspects** |  |  |
| It would be a sport predominantly for boys | 6 | 0.8% |
| it's not a sport | 33 | 4.3% |
| inaccessible sports/closed circle only for elites | 24 | 3.1% |
| That they are wasting their time/ that they are good for nothing in real life | 17 | 2.2% |
| That it is not a big deal to play chess | 1 | 0.1% |
| That it is not an interesting/boring sport | 5 | 0.6% |
| that you cannot perform well in chess in Romania/Romanians are not good chess players | 8 | 1.0% |
| That you can cheat with electronic means | 1 | 0.1% |
| **Structures that take care of chess** |  |  |
| Lack of help from the competent/financing structures | 7 | 0.9% |
| Club membership fees are high | 1 | 0.1% |
| **Individual aspect** |  |  |
| reduced physical abilities/predisposition to sedentarism | 39 | 5.0% |
| School absenteeism during competitions | 3 | 0.4% |
| I don't know anything else except chess | 1 | 0.1% |
| financial rewards reduced/not commensurate with investments | 27 | 3.5% |
| Arrogance | 18 | 2.3% |
| cause a decrease in social skills | 1 | 0.1% |
| psychologically aggressive | 1 | 0.1% |
| overrated benefits | 2 | 0.3% |
| difficult | 1 | 0.1% |
| it's only for smart/intelligent/genius people | 10 | 1.3% |
| that it is the sport of misfits, antisocials, "nerds", weird/individualists/autists | 57 | 7.4% |
| That it is an individual sport and that it would not promote team spirit | 1 | 0.1% |
| That I think too much | 3 | 0.4% |
| That they don't have motor intelligence (false by the way) | 1 | 0.1% |
| Because I don't know anything else | 2 | 0.3% |
| Because chess players are too serious | 1 | 0.1% |
| Training takes time | 2 | 0.3% |
| That they specialize in a single discipline, trying to be good. There are much more complex disciplines | 2 | 0.3% |
| That they are animated only by the desire to win | 1 | 0.1% |
| That they are selfish. | 3 | 0.4% |
| That they are geniuses. And in fact, they are only people with a very good memory and with many logical schemes memorized. | 1 | 0.1% |
| That they are stubborn | 1 | 0.1% |
| That they are introverts | 17 | 2.2% |
| That they are forced by their parents to participate in contests and that they don't have time for other games | 2 | 0.3% |
| That you must have talent to play chess | 1 | 0.1% |
| Ability to concentrate | 2 | 0.3% |
| maturity | 1 | 0.1% |
| The biggest stupidity - who is exempt from physical education can go play chess (your ace in the case of children). | 1 | 0.1% |
| The competition | 1 | 0.1% |
| Unpopular competitions | 1 | 0.1% |
| Costs too high for parents | 3 | 0.4% |
| Poor communication | 13 | 1.7% |
| The nervous consumption during competitions is too high for children | 2 | 0.3% |
| I think they are respected | 1 | 0.1% |
| if I play chess, I develop faster | 1 | 0.1% |
| memory development | 1 | 0.1% |
| Distance from other sports | 1 | 0.1% |
| It takes exceptional potential to play chess | 1 | 0.1% |
| Selfishness | 6 | 0.8% |
| It's hard | 6 | 0.8% |
| Women do not have the same results as men | 1 | 0.1% |
| creationist | 1 | 0.1% |
| The degree of difficulty of the workouts | 1 | 0.1% |
| plump | 2 | 0.3% |
| individualist | 15 | 1.9% |
| envy | 3 | 0.4% |
| Lack of money | 24 | 3.1% |
| Lack of other procurements | 2 | 0.3% |
| Lack of social appreciation | 1 | 0.1% |
| Lack of audience | 1 | 0.1% |
| Lack of sports clubs in cities | 1 | 0.1% |
| Lack of horizons after finishing junior studies | 10 | 1.3% |
| Lack of immediate results | 1 | 0.1% |
| Ignorance of the benefits of chess | 7 | 0.9% |
| frivolity | 1 | 0.1% |
| They have no imagination | 1 | 0.1% |
| No time for performance chess because of school | 2 | 0.3% |
| Takes up too much free time for preparation | 12 | 1.6% |
| People with patience of steel. | 1 | 0.1% |
| Concentrated power and duration. | 1 | 0.1% |
| Stiffness. | 1 | 0.1% |
| Chess players waste too much time in front of the computer | 1 | 0.1% |
| retirees | 3 | 0.4% |
| chess does not bring benefits in the short term, so it is not worth so much mental and financial effort | 1 | 0.1% |
| Chess only involves science without emotion | 1 | 0.1% |
| It shortens the time for lessons | 1 | 0.1% |
| It is believed that they have no social life | 1 | 0.1% |
| Too many analysis engines are used, chess has lost its authenticity | 1 | 0.1% |
| They are idealists | 3 | 0.4% |
| They are too obsessed with competition results | 2 | 0.3% |
| They are only concerned with chess | 1 | 0.1% |
| They are seen as people with dull thinking | 1 | 0.1% |
| overrate | 1 | 0.1% |
| Suspicious | 1 | 0.1% |
| mathematical skills | 1 | 0.1% |
| NA | 230 | 29.7% |
| Total | 774 | 100% |

**Table 1.d. q30. Describing the parents who have children at** chess classes**(open-ended)**

|  | Count | Percentage |
| --- | --- | --- |
| involved in children's education | 92 | 11.9% |
| Ambitious | 18 | 2.3% |
| adapter | 2 | 0.3% |
| activity | 2 | 0.3% |
| Anchored in reality | 1 | 0.1% |
| patient | 3 | 0.4% |
| Pay attention to the child's needs | 34 | 4.4% |
| They trust their own children | 8 | 1.0% |
| Well intentioned | 2 | 0.3% |
| adventuress | 1 | 0.1% |
| calculated/calm/balanced | 6 | 0.8% |
| Good mobilisers | 3 | 0.4% |
| good | 2 | 0.3% |
| Organizational capacity | 14 | 1.8% |
| Strong character | 2 | 0.3% |
| Competitive | 7 | 0.9% |
| Compromise | 1 | 0.1% |
| You are aware of the advantages of this activity | 4 | 0.5% |
| Cooperation | 1 | 0.1% |
| correction | 1 | 0.1% |
| With the desire to surpass oneself | 5 | 0.6% |
| With the initiative | 2 | 0.3% |
| With long-term goals | 1 | 0.1% |
| With flexible schedule | 2 | 0.3% |
| With similar value systems | 1 | 0.1% |
| courageous | 5 | 0.6% |
| Cultured/educated/intellectuals | 15 | 1.9% |
| Sports connoisseurs in general | 1 | 0.1% |
| Curious | 6 | 0.8% |
| open | 28 | 3.6% |
| Smart | 12 | 1.6% |
| Disciplined. | 3 | 0.4% |
| Willing to offer in advance and get involved in the children's activity, financially | 56 | 7.2% |
| Devotion | 1 | 0.1% |
| Willing to make sacrifices | 2 | 0.3% |
| They think about the children's future | 4 | 0.5% |
| caring | 2 | 0.3% |
| emancipated | 1 | 0.1% |
| efficiency | 2 | 0.3% |
| Learn about the benefits of chess, education, etc | 21 | 2.7% |
| breathe | 55 | 7.1% |
| Understandable | 5 | 0.6% |
| intelligent | 36 | 4.7% |
| interconnected | 1 | 0.1% |
| intuition | 4 | 0.5% |
| They want something special for their children | 1 | 0.1% |
| lovers | 1 | 0.1% |
| lucid | 3 | 0.4% |
| consistently | 1 | 0.1% |
| More informed than the rest | 8 | 1.0% |
| Proud of their children | 4 | 0.5% |
| Behavior models for their children | 4 | 0.5% |
| lovely | 5 | 0.6% |
| ordinary | 28 | 3.6% |
| Lucky | 6 | 0.8% |
| occupy | 1 | 0.1% |
| optimistic | 4 | 0.5% |
| conceited | 1 | 0.1% |
| responsible | 89 | 11.5% |
| persevering | 11 | 1.4% |
| passionates | 1 | 0.1% |
| pragmatically | 3 | 0.4% |
| cautious | 1 | 0.1% |
| patient | 23 | 3.0% |
| reason | 2 | 0.3% |
| realistically | 4 | 0.5% |
| They think about the future | 1 | 0.1% |
| Serious | 2 | 0.3% |
| Watchers | 5 | 0.6% |
| vain | 1 | 0.1% |

**Table 2.a.** Independent Samples Test group of degree-positive aspects

|  |  |  |  | | t-test for Equality of Means | | | | | | |
| --- | --- | --- | --- | --- | --- | --- | --- | --- | --- | --- | --- |
|  | Group | N | Mean | S. D. | t | df | p | Mean Difference | Std. Error Difference | CI4 | |
|  | Do you know to play chess |  |  |  |  |  |  |  |  | Lower | Upper |
| 2. Competitive spirit | Yes | 513 | 3.57 | 0.58 | 5.41 | 423 | 0.000 | 0.28 | 0.05 | 0.18 | 0.39 |
|  | No | 257 | 3.28 | 0.74 |  |  |  |  |  |  |  |
|  | Total | 770 | 3.48 | 0.65 |  |  |  |  |  |  |  |
| 3. Spirit of cooperation | Yes | 507 | 3.15 | 0.76 | 3.83 | 760 | 0.000 | 0.22 | 0.05 | 0.11 | 0.34 |
|  | No | 255 | 2.93 | 0.76 |  |  |  |  |  |  |  |
|  | Total | 762 | 3.08 | 0.77 |  |  |  |  |  |  |  |
| 6. Mental abilities | Yes | 515 | 3.85 | 0.38 | 2.57 | 473 | 0.010 | 0.08 | 0.03 | 0.01 | 0.14 |
|  | No | 256 | 3.77 | 0.42 |  |  |  |  |  |  |  |
|  | Total | 771 | 3.82 | 0.40 |  |  |  |  |  |  |  |
| 7. Emotional capacities | Yes | 510 | 3.43 | 0.64 | 2.19 | 764 | 0.029 | 0.10 | 0.05 | 0.01 | 0.20 |
|  | No | 256 | 3.32 | 0.65 |  |  |  |  |  |  |  |
|  | Total | 766 | 3.39 | 0.65 |  |  |  |  |  |  |  |
| 8. Strong character | Yes | 509 | 3.50 | 0.62 | 5.11 | 761 | 0.000 | 0.25 | 0.04 | 0.15 | 0.35 |
|  | No | 254 | 3.25 | 0.68 |  |  |  |  |  |  |  |
|  | Total | 763 |  |  |  |  |  |  |  |  |  |
| 9. Self-discipline | Yes | 511 | 3.44 | 0.68 | 4.92 | 763 | 0.000 | 0.26 | 0.05 | 0.16 | 0.37 |
|  | No | 254 | 3.17 | 0.75 |  |  |  |  |  |  |  |
|  | Total | 765 | 3.42 | 0.65 |  |  |  |  |  |  |  |
| 10. Ability to make quick decisions | Yes | 514 | 3.38 | 0.77 | 6.29 | 765 | 0.000 | 0.38 | 0.06 | 0.26 | 0.51 |
|  | No | 253 | 2.99 | 0.86 |  |  |  |  |  |  |  |
|  | Total | 767 | 3.25 | 0.82 |  |  |  |  |  |  |  |
| 11. High self-esteem | Yes | 509 | 3.37 | 0.70 | 5.33 | 762 | 0.000 | 0.29 | 0.05 | 0.18 | 0.40 |
|  | No | 255 | 3.07 | 0.76 |  |  |  |  |  |  |  |
|  | Total | 764 | 3.27 | 0.73 |  |  |  |  |  |  |  |
| 12. Inuition | Yes | 511 | 3.48 | 0.70 | 5.53 | 764 | 0.000 | 0.31 | 0.05 | 0.20 | 0.42 |
|  | No | 255 | 3.17 | 0.79 |  |  |  |  |  |  |  |
|  | Total | 766 | 3.38 | 0.747 |  |  |  |  |  |  |  |
| Q9 index positive aspects | Yes | 515 | 3.37 | 0.39 | 5.79 | 771 | 0.000 | 0.17 | 0.02 | 0.11 | 0.23 |
|  | No | 258 | 3.20 | 0.38 |  |  |  |  |  |  |  |
|  | Total | 773 | 3.34 | 0.40 |  |  |  |  |  |  |  |
| Q20.  Chess helps the children to develop positive emotions | Yes | 516 | 4.45 | 0.76 | 3.63 | 772 | 0.000 | 0.21 | 0.05 | 0.09 | 0.33 |
|  | No | 258 | 4.24 | 0.80 |  |  |  |  |  |  |  |
|  | Total | 774 | 4.38 | 0.78 |  |  |  |  |  |  |  |
| Q18. Chess helps the children to overcome the negative emotions | Yes | 516 | 4.26 | 0.85 | 2.96 | 772 | 0.003 | 0.19 | 0.06 | 0.06 | 0.31 |
|  | No | 258 | 4.07 | 0.81 |  |  |  |  |  |  |  |
|  | Total | 774 | 4.20 | 0.84 |  |  |  |  |  |  |  |

**Table 2.b.** Independent Samples Test group of degree-negative aspects

|  |  |  |  | | t-test for Equality of Means | | | | | | |
| --- | --- | --- | --- | --- | --- | --- | --- | --- | --- | --- | --- |
|  | Group | N | Mean | S. D. | t | df | p | Mean Difference | Std. Error Difference | CI4 | |
|  | Do you know to play chess |  |  |  |  |  |  |  |  | Lower | Upper |
| 10. Addiction to adrenaline from competition | Yes | 486 | 1.41 | 0.67 | 2.85 | 593 | 0.004 | .013 | 0.04 | 0.04 | 0.22 |
|  | No | 251 | 1.27 | 0.56 |  |  |  |  |  |  |  |
|  | Total | 737 | 1.36 | 0.64 |  |  |  |  |  |  |  |
| 12. Negative impact on mental health | Yes | 488 | 1.12 | 0.41 | 2.69 | 717 | 0.007 | 0.06 | 0.02 | 0.01 | 0.11 |
|  | No | 245 | 1.05 | 0.24 |  |  |  |  |  |  |  |
|  | Total | 733 | 1.10 | 0.37 |  |  |  |  |  |  |  |
| Q10 index negative aspects | Yes | 509 | 1.30 | 0.41 | 0.93 | 651 | 0.35 | 0.02 | 0.02 | -0.02 | 0.07 |
|  | No | 257 | 1.34 | 0.31 |  |  |  |  |  |  |  |
|  | Total | 716 | 1.36 | 0.38 |  |  |  |  |  |  |  |
